# Consumers’ Attitudes and Purchase Intention for a Vitamin-Enriched Extra Virgin Olive Oil

**DOI:** 10.3390/nu14081658

**Published:** 2022-04-15

**Authors:** Manal Hamam, Giuseppe Di Vita, Raffaele Zanchini, Daniela Spina, Maria Raimondo, Manuela Pilato, Mario D’Amico

**Affiliations:** 1 Department of Agriculture, Food and Environment (Di3A), University of Catania, 95123 Catania, Italy; manal.hamam@phd.unict.it (M.H.); mario.damico@unict.it (M.D.); 2 Department of Agricultural, Forest and Food Science (Disafa), University of Turin, Largo Braccini, 10095 Grugliasco, Italy; giuseppe.divita@unito.it (G.D.V.); raffaele.zanchini@unito.it (R.Z.); 3 Department of Agricultural Sciences, University of Naples Federico II, 80055 Portici, Italy; maria.raimondo@unina.it; 4 Department of Marketing, Events Management, and Project Management, University of Winchester, Winchester SO22 5HT, UK; manuela.pilato@winchester.ac.uk

**Keywords:** consumers’ preferences, vitaminized olive oil, willingness to pay, premium price, functional foods, healthy olive oil

## Abstract

This study aims to examine Italian consumer preferences for extra virgin olive oil (EVOO) enriched with vitamins and to analyze the key drivers that affect consumer choices for this product. Specifically, we assessed consumers’ intention to purchase the enriched product compared to the conventional one. The methodology adopted inferential and multivariate statistical techniques: (1) exploratory factor analysis (EFA), (2) ordinary least squares regression (OLS) and (3) non-hierarchical clustering. This study appears to be the first research project related to exploring consumers’ interest in an extra virgin olive oil enhanced with vitamins, thereby providing preliminary indications. The main results represent a significant starting point for the development of new marketing strategies for the food industry.

## 1. Introduction

Foods enhanced with vitamins, minerals or other constituents are becoming increasingly popular and they are gaining more and more ground in trade and consumption, with previous studies exploring the existence of health motivations as drivers for this growth [1,2,3]. Further research confirms this tendency, and identifies the healthiness attribute as one of the crucial factors in influencing consumers’ behavior, especially in European countries [4,5,6].

In fact, health is one of the major concerns that affect consumers’ lives, and its relevance in influencing purchasing behavior is expanding, particularly in regard to food choices [1,7,8]. As a result, consumers’ attention has also shifted to aspects related to the food–health connection [9,10]. Consumers no longer consider food only as a means of meeting their nutritional needs, but also as a tool capable of preventing and reducing the risk of diseases [11,12] such as obesity and diabetes that are on the rise around the world [13,14], and a way to improve life quality and to achieve a longer life expectancy [15].

As a result, non-price characteristics have become increasingly important in food purchasing choices [16]. Trends reveal that people tend to highly value foods with natural features and health claims and are willing to pay an additional premium price for these attributes [17].

In order to meet the demand for healthier foods, companies need to offer products with a natural image or emphasize their health benefits [18]. The latter include fortified or enriched products [19,20].

Fortified foods are a subcategory of functional foods with additional constituents, such as vitamins, fiber or minerals [21], to improve the health benefits and to increase intake levels in specific demographic groups, for example, infant formula. The fortification of food began as a process used to restore any losses that occurred during the production process. However, nowadays, the food industry’s objective is to increase the sales and consumption of enriched products by focusing on their potential beneficial effects on health [22,23].

Enriched foods are regulated by the European Regulation (EC) No. 1925/2006 [24], which establishes a list of allowed substances as well as their sources. These regulations are the result of a lengthy process, with the aim to regulate and harmonize the legislation regarding fortification in Europe. The rules state that in addition to classifying the permitted limits of the nutrients to be added, a nutritional table containing the values of the supplemental nutrients must be included in the product packaging. At the turn of the 21st century, the Scientific Committee on Food (SCF) began issuing guidelines to define the upper limits (UL) for the intake of vitamins and minerals in food.

Within this novel regulatory framework, the European Food Safety Authority (EFSA) classified olive oil as a functional food since it possesses nutritional benefits that occur naturally, it is rich in healthy compounds [25], and it experiences progressive changes in its composition. Among the health components found in olive oil are polyphenols, particularly hydroxytyrosol and its derivatives (oleuropein and tyrosol complex), which aid in the protection of blood lipids against oxidative stress.

It is worth noting that the significant improvement in extra virgin olive oil (EVOO) nutritional characteristics, as well as a deep transformation in its sensory profile, have converted a traditional product with well-recognized organoleptic properties, into a novel condiment [26].

In this context, large olive oil companies, which have opted for a qualitative leap, are aware that the production of an enriched olive oil is an approach that boosts its benefits and opens up new markets and new business opportunities [25]. Innovative products have already emerged in the olive industry and are becoming progressively more widespread. In this regard, two different studies have addressed the topic from a technological and nutritional perspective; the first one addressed the production feasibility of a EVOO enriched with lycopene, which enhances the antioxidant properties with beneficial health effects for humans [27]. In the other study, Limón et al. [28] demonstrated the beneficial effects of enriched olive oils on health by enhancing the carotenoids content in the EVOO. This evidence showed that enriched olive oil might be more effective in the prevention of diseases connected to visual degeneration.

The medical and nutritional literature stresses the importance of numerous beneficial effects of extra virgin olive oil on human health, especially considering that it is rich in vitamin E and has both antioxidant and anti-inflammatory properties. However, to the best of our knowledge, it seems that there are no previous studies that have explored consumers’ interest in an extra virgin olive oil enhanced with vitamins. Most of the scientific literature on consumers’ preferences is limited to functional foods that are enriched with micronutrients [29]. In addition, there are some studies on vitamin-enriched foods [30,31]. Surai and Sparks [32], for example, have shown that eggs can be used as functional foods after enrichment with micronutrients, including vitamin D, E and B12. In particular, there is strong evidence that vitamin D reduces the risk of osteoporosis [33,34] while vitamin E is a protective antioxidant micronutrient that has a role in the prevention of atherosclerosis and carcinogenesis, and in reducing the risk of cardiovascular disease [35,36].

However, there is a lack of research on consumers’ attitudes and preference for a vitamin-enriched olive oil. Based on this premise, the present study appears to be the first to analyze Italian consumer preferences for a vitaminized extra virgin olive oil and it aims to explore the key drivers that may influence purchase intention for this product.

To this purpose, we examined the data through inferential and multivariate statistical and econometric techniques. A three-step approach has been adopted: an exploratory factor analysis (EFA), an ordinary least squares regression (OLS), and a non-hierarchical cluster analysis. This work addresses the following research questions:

1. Which drivers can be associated with vitamin-enriched olive oil purchases?
2. Which factors affect consumers’ willingness to buy for vitamin-enriched olive oil?
3. What are the most relevant demographic characteristics of vitaminized olive oil consumers?

## 2. Material and Methods

### 2.1. Data Collection and Conceptual Framework

The data collection took place in Italy in May and June 2020. To overcome the difficulty of reaching a geographically-dispersed population, the sampling technique chosen was snowball sampling [37], i.e., a targeted, exponential, non-discriminatory, non-probabilistic sampling technique, also known as “chain referencing”, which acquires data through existing social structures, whereby each study subject recruits future subjects from among their acquaintances [38]. It consists of contacting a small sample from the target subpopulation (such as direct acquaintances), who are asked to involve other participants in the study, as in a kind of chain reaction. Usually, this technique is used to explore topics that are difficult to investigate directly or publicly (such as drug use); however, where there is no need for a probabilistic population sample, snowball sampling, together with the use of social networks, allows the researcher to quickly and exponentially reach many users, and in this case consumers. The communication channels used were social networks, i.e., the virtual platforms currently most in use.

A multi-section questionnaire using Google Forms and shared online in several social networks and specialized forums was used to acquire data anonymously. The questionnaire was divided into three sections: sociodemographic characteristics (age, gender, and education); generalities concerning the consumption of extra virgin olive oil; and interest and purchase intention for an olive oil enriched with vitamin D and E compared to a conventional one.

The section called “generalities of olive oil consumption” included questions related to the safety and quality of olive oil, intrinsic and extrinsic attributes and health-related aspects of consumption.

The questions were organized either as binary questions with yes or no answers, for example: “Do you regularly buy extra virgin olive oil?” or using a seven-point Likert scale. Some examples of the latter are: “In your opinion, how safe is extra virgin olive oil?” or “How useful do you think the consumption of extra virgin olive oil is for the improvement of your health and in the prevention of some diseases?” where 1 = not at all and 7 = a lot, or “Could you please indicate the level of importance about the reasons why you consume Extra Virgin Olive Oil?” where 1 = less relevant and 7 = more relevant. Finally, the interest in vitamin-enriched olive oil was addressed using binary questions, while the additional premium price was addressed using a question where seven alternatives were provided ranging from not willing to pay to willing to pay over 50% more. Each level of the additional premium price will be explained further in the data analysis subsection.

The collected sample consisted of 588 individuals whose characteristics, gender, age and education are presented in Table 1. With regard to age classes, a brief explanation of the categorization is required. Starting from the age classification provided by Brosdahl and Carpenter [39], the authors focused on the following two categories: younger generations, including Millennials (1982–2000) and Generation Z [40]; and Generation X (1961–1981).

### 2.2. Data Analysis

The survey aimed to evaluate the relevance that the intrinsic and extrinsic attributes of extra virgin olive oil enriched with vitamin E and D have on consumers’ intention to buy, thus evaluating their willingness to pay a premium price. The data collected were analyzed through inferential and multivariate statistical techniques. Three models were used for this purpose: EFA, OLS and non-hierarchical clustering. In this approach, EFA represents the preliminary step to be carried out; the results are then used in the regression as predictors and in the cluster analysis as differentiation variables in order to associate consumers’ characteristics with interest in functional and vitamin-enriched olive oil.

#### 2.2.1. Factor Analysis

Factor analysis was performed firstly to reduce the number of variables, and thus to produce new factorial dimensions in which the original predictors are merged, minimizing the loss of variance according to the correlation matrix [41]. The factor analysis permitted us to summarize the information highlighting the latent relations among the original variables; the factors produced can be used in other analysis such as regressions [42] or cluster analysis [43]. The factor produced can be considered orthogonal since the EFA was conducted using principal component analysis [42]. Varimax rotation was applied to maximize the sum of the variance of the square loadings, which also permits us to simplify the interpretation of the Principal Component Analysis (PCA) [44]. The selection of the number of factors was based on the rule of an eigenvalue greater than 1 [45] and coefficients for the interpretation over 0.6, in order to obtain a robust assessment [46].

Two tests were performed: the Kaiser–Meyer–Olkin (KMO) and Bartlett’s test in order to assess the goodness of fit of the exploratory factor analysis. The first test compares the observed correlations and partial correlations between the variables employed in the model, producing an index with values between 0 and 1. To consider the reliability of the factor analysis as good, the results of the KMO test should be higher than 0.7. Indeed, an index lower than 0.5 indicates the inadequacy of the factorial model because the correlation between pairs of variables can not be described by the variance shared by the whole group of variables [47]. The Bartlett’s test was used to test the null hypothesis that the correlation matrix coincides with the identity matrix, assessing whether the total correlation of the model is 0. Therefore, to consider the analysis reliable, the test result must be significant as this indicates that the coincidence between the matrices was observed.

#### 2.2.2. OLS Regression

Then, to assess the significance of each factor obtained by the EFA, an OLS regression was created, represented by the following relationship:

WTP = *β*_1_ + *β*_2_*x*_Age_ + *β*_3_*x*_Gender_ + *β*_4_*x*_Education_ + *β*_5_*x*_Disease Prevention_ + *β*_6_*x*_Perception_ + *β*_7_*x*_Intrinsic Factors_ + *β*_8_*x*_Extrinsic Factors_ + *β*_9_*x*_Beliefs_ + *β*_10_*x*_Organoleptic Qualities_ + *β*_11_*x*_Purchasing Behavior_ + ε_*i*_ (1)

where: WTP, willingness to pay; *β*_1–11_, slope; *x*, independent variable (predictor); ε*i*, error term.

The dependent variable, WTP is represented by the willingness to pay more than the average purchase price for a functional oil enriched in vitamins, categorized as: No willingness to pay 10%; 20%; 30%; 40%; 50%; >50%. The regressors included the sociodemographic variables, age (continuous), gender (dummy) and education (categorical) and the variable, disease prevention (dummy), which is represented by “how useful do you think the consumption of extra virgin olive oil is for the improvement of your health and in the prevention of some diseases?”.

In addition, all six factors of the EFA were included in the regression. The advantages of adopting the factors previously obtained in the regression model are two-fold: the system is simplified due to dimensional reduction and collinearity is avoided because the factors are orthogonal. The new factorial dimensions used in the OLS were: perception, that is, the level of importance that the consumer attributes to extra virgin olive oil in regard to its wholesomeness, digestibility, nutritional properties and culinary traditions; intrinsic factors, which provide information about the internal attributes of the product including safety, quality, color, taste, aroma and consistency; and extrinsic factors, which provide information about the external attributes of the product.

The extrinsic factors evaluated in this study were the following: the brand, price, label indications, the origin of the olives, quality certifications, and traceability. Other factors include beliefs, i.e., how much the consumer believes that extra virgin olive oil compared to a seed oil is less fatty, contains no additives, helps to maintain low cholesterol and is less harmful; the organoleptic qualities, i.e., how much the bitter and spicy flavors, according to the consumer, define the quality of an extra virgin olive oil; and finally, purchasing behavior, i.e., whether regular extra virgin olive oil is purchased and how many times.

#### 2.2.3. Cluster Analysis

Factorial dimensions were also used to cluster consumers and obtain homogeneous groups using the non-hierarchical k-mean method [48]. The method permits the development of groups by iterative procedures, minimizing the Euclidean distances between group’s centroids [49]. 

**Figure A1:**
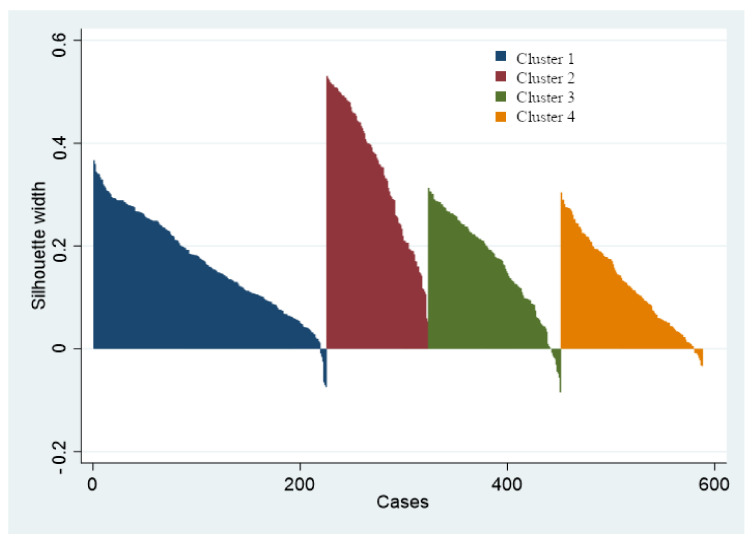
Silhouette results for the best cluster solution.

In cluster analysis, the issue of the best cluster solution should be considered, especially in the case of non-hierarchical methods where the formation of clusters is chosen a priori [50]. In order to deal with this aspect, firstly, silhouette width was chosen as an indicator of cluster adequacy [51]. Several cluster solutions were tested and through the interpretation of the graphs, the four-cluster solution was found to be the most suitable; the graph of the best solution can be found in Appendix A (Figure A1). Secondly, to check the significant differences among the best cluster solution score, one-way ANOVA was performed [52]. Finally, to verify the association among the frequencies of categorical variables such as socio-demographic variables and interest in olive oil enriched with vitamins among clusters, chi-square tests were performed [53]. All analyses were performed using Stata software 17.0 Standard Edition (StataCorp LLC., College Station, TX, USA).

## 3. Results

The results are organized in three different steps: exploratory factor analysis, willingness to buy and cluster analysis, in order to assess the main attributes and characteristics linked to a vitaminized olive oil and to build a socio-economic profile of consumers potentially interested in buying a functional olive oil.

(a) Exploratory factor analysis

By means of the EFA application, we proceeded to create six factors as shown in Table 2: perception, intrinsic factors, extrinsic factors, beliefs, organoleptic qualities and buying habits.

The orthogonal rotation approach, with factor loading values greater than 0.6, was used to represent the strength of the relationship between the observable variables, which all had values between 0.7 and 0.8, and the factors. On the other side, the beliefs and perceptions components elicit information regarding the health and nutritional benefits of olive oil. Bitter and spicy were used to characterize the organoleptic qualities, as these attributes are known to be essential in the WTP of high-end oils, whereas factors relating to oil consumption frequency were used to define the purchasing behavior component.

The results show us that the goodness of fit indices of the model validate the reliability of the exploratory factor analysis. In fact, in Bartlett’s test of sphericity, the *p*-value turns out to be highly significant (0.000 ***), i.e., we reject the hypothesis of correlation between the variables being significantly different from zero and accept the hypothesis of correlation between them. The KMO test also confirms the above. Again, the value of 0.899 confirms the reliability of the exploratory factor analysis.

(b) Willingness to buy a functional olive oil

The second step of our study evaluated the willingness to buy a vitamin-enriched olive oil. By analyzing the descriptive statistics, our results revealed that 65% of the sample analyzed is willing to pay more for a functional extra virgin oil. Specifically, more than half (53%) are willing to pay from 10% to 30% more, while 12% are willing to pay up to 40% or more. However, a high percentage of the sample (35%) are not willing to pay a higher price for a vitamin-enriched olive oil than for a conventional oil, probably because the extra virgin oil is already perceived by consumers as a functional product that is naturally characterized by excellent nutritional and healthy properties.

To assess the significance of each factor, an OLS regression was carried out. Table 3 shows the results of the OLS regression whereby the dependent variable is represented by WTP. It was found for the observed sample that as age increases, the willingness to pay for a vitaminized olive oil decreases (*p* <0.001 ***), while believing that consumption of extra virgin olive oil can prevent disease increases the willingness of consumers to pay more (*p* = 0.001 **). Extrinsic factors (*p* = 0.070 **) and purchasing behavior (*p* = 0.080 *) were also shown to be positively correlated with willingness to pay, while the perception variable appeared to be negatively correlated (*p* = 0.078 *).

(c) Cluster analysis

The results of k-clustering led to the creation of four clusters, each distinguished by different characteristics of individuals in the cluster. As shown in Table 4, consumers in the first cluster, consisting of 225 individuals, have positively expressed purchasing behavior and pay much attention to the oil’s organoleptic qualities, specifically, the bitter and spicy flavor. Moreover, these consumers, who are regular buyers of extra virgin olive oil, consider this product as typical of the culinary tradition, and consequently they perceive it as a healthy and genuine food with excellent nutritional properties. Based on these characteristics, the group was defined as “aware healthy consumers”. In terms of socio-demographic features (Table 5), this first cluster is mainly characterized by women, with a high level of education and are interested in purchasing functional vitaminized olive oil.

In the second cluster, made up of 98 individuals, the factor related to purchasing behavior is slightly positively expressed, while all other factorial dimensions are negatively expressed. This information suggests that consumers in this cluster purchase olive oil for consumption, as a cooking or seasoning food, without paying particular attention to the intrinsic, extrinsic, or health characteristics of the product. For these reasons the cluster was called “skeptical consumers”. Compared to the first cluster, the number of males is greater and the level of education is medium to high (Table 5).

The third cluster, composed of 128 individuals, negatively expresses the factor related to frequency of consumption. This information suggests that the individuals that belong to this cluster are occasional consumers. This can be presumed from the fact that the other factorial dimensions are expressed positively, for example, organoleptic qualities and beliefs, even though their weight is not higher or lower than the same factorial dimensions in the other clusters. For these reasons, the group was called “occasional consumers”. Considering the sociodemographic characteristics, this cluster comprises mainly young individuals with a medium to high level of education and this group is less interested in buying a functional extra virgin olive oil.

The fourth cluster, made up of 137 individuals, is comprised of those interested in the intrinsic and extrinsic attributes of the product and who believe that consuming an extra virgin olive oil is less harmful than consuming a seed oil. In fact, the factors linked to these variables are positively marked by higher scores than the other clusters. For these reasons, the group was defined as the “informed quality seekers”. In this cluster, women, individuals belonging to the Generation X and elders are the most represented classes; in addition, we even find consumers with primary and middle school education, probably due to the higher presence of older consumers.

## 4. Discussion

Extra virgin olive oil is a healthy, traditional food product [54,55] that is closely linked to the gastronomic heritage of the Mediterranean countries and is characterized by minimal processing. Thousands of people recognize EVOO and consume it just the way it is. Therefore, the findings of our study highlight something quite new: consumer willingness to pay more for vitamin-enriched extra virgin olive oil. This result is more than interesting considering that the consumers in our sample live in a country, Italy, where olive oil is part and parcel of the gastronomic tradition. Although food naturalness is very important for consumers [56,57], the word “vitamin” sounds familiar to them and it is one they connect to health benefits.

Our research has specifically analyzed the features that steer consumers towards a vitamin-enriched oil (factor analysis), the factors that affect consumer’s willingness to pay an additional price premium for it (OLS regression), and the main socio-demographic characteristics associated with an interest in functional and vitamin-enriched olive oil (cluster analysis). Since most products that integrate innovations fail to succeed in the market [58], research suggests taking the consumers’ point of view into account to stop new products failing and the subsequent waste of resources [59]. This represents the first investigative step in understanding how consumers will react to a vitaminized olive oil.

The exploratory factor analysis has shown what drives consumers towards vitamin-enriched olive oil. The perception linked to healthiness, digestibility, nutritional properties and culinary traditions is crucial in the decision to consume extra virgin olive oil, as highlighted by other studies [6,60,61]. For example, Delgado and Guinard [62] reported that 74% of a US sample indicated health benefits as the main motivation for consuming extra virgin olive oil. Peršuric and Damijanic [63] confirmed that the intention to consume olive oil was deeply equated with health benefits. Our results, in fact, show that most consumers are aware of the positive associations between health and olive oil consumption, especially woman who prioritize healthy eating.

Moreover, it is interesting to note the role that the organoleptic qualities play, specifically, bitter and spicy. As the results show, bitterness and spiciness, which represent those attributes that determine the quality of an extra virgin olive oil [64], have a positive influence on consumer interest in a vitamin-enriched extra virgin olive oil. This outcome is quite new in the scientific literature. Generally, consumers do not tend to favor bitterness in food [65]. Yet, when speaking about extra virgin olive oil, bitterness is fundamental because it signifies healthiness, being closely linked to antioxidant content, which helps protect human cells from free radicals [66,67,68]. Although sufficient evidence exists to verify that extra virgin olive oils with a pungent flavor have a higher polyphenol content (hydroxytyrosol, oleuropein and tyrosol), that is, healthier properties [69,70], most consumers do not seem to prefer bitter-tasting food products [71,72,73].

With regard to the sample examined in our study, based on the OLS regression results, we can state that the extra price percentage that customers are willing to pay for a vitaminized extra virgin olive oil is influenced by its role in disease prevention, confirming that health concerns are always an important purchasing incentive, as well as extrinsic factors that are confirmed as decisive variables in marketing strategies [73] and purchasing behavior. The latter is positively correlated with the willingness to pay an additional price premium.

Our study also examined the attributes affecting olive oil consumer preferences. In our model, brand, price, label indication, olive provenance, quality certification and traceability emerged as the most significant extrinsic characteristics. All the outcomes are widely confirmed by previous literature, for example, brand has a significant impact on the perceived quality of a product [74], and as a result, it positively influences the willingness to buy [26]. It plays a crucial role, especially in the marketing of large-scale distribution of extra virgin olive oil [75]. Price is a significant factor in purchasing decisions and is among the extrinsic attributes that have the greatest impact on willingness to buy [76]. Previous studies show that the consumer considers price an important quality index [77]. Ballco et al. [78] examined the willingness to pay for extra virgin olive oil, finding that price is the most influential extrinsic attribute in the purchase decision [79,80,81] followed by the origin and the Protected Designation Origin (PDO) certification [82,83]. Among the extrinsic attributes, it is worth noting the role that food labels play, particularly nutritional ones, in directing consumers towards healthy food choices [84,85,86]. Our findings confirm that food acceptance is influenced by the information on the label about the origin of the product, quality, composition and the production process [72], so, the food label influences the perception of the taste and quality of a product, and hence the intention to purchase it. The growing interest in functional foods has led several authors to pay attention to labels with nutritional claims to evaluate whether they might help consumers identify functional foods in a better way [84,85]. In fact, research carried out by Roselli et al. [87] has shown that the indications relating to the polyphenol content of olive oil help to emphasize the beneficial qualities of extra virgin olive oils. However, although it has been proven that information regarding the nutritional content of foods strongly influences consumer choices [88], this nutritional information is frequently misunderstood by consumers due to a lack of objective knowledge [85,89,90].

The results of our study also evidenced how the traceability of extra virgin olive oil plays an essential role in the purchase decision. Indeed, as previous studies have shown [91,92], food safety provides a justification for the growing willingness to pay for food traceability. However, even though traditional communication channels are vital for transmitting information, consumers today also seek rational support for their purchasing decisions [93], which is why a better traceability system can be considered an interesting response to consumer needs [94].

Furthermore, several studies reflect the growing desire among consumers to be involved in food production, quality and traceability [95,96]. Information and knowledge create awareness, which in turn, can increase the willingness of consumers to pay for local extra virgin olive oil [97,98,99].

The high frequency of extra virgin olive oil purchasing is another factor that influences the intention to buy a vitamin-enriched extra virgin olive oil. Those consumers who associate olive oil with health are likely to buy more of this product.

Finally, as shown in the cluster analysis, we identified four classes of consumers, with different levels of interest in vitaminized olive oil or even disinterest. The results of our research show that high consumer interest in the intrinsic and extrinsic characteristics is mainly found in the quality seekers cluster, predominantly made up of women, Generation X individuals, and the elderly, who showed an interest in vitamin-enriched olive oil. We noticed that female gender and high education are the variables associated with consumer interest in the functional properties of olive oil as in the case of healthy consumers, thus confirming that consumers’ perception towards vitamin-enriched food is positively correlated with high education [100,101].

On the other side, we discovered two classes of consumers partially or not interested at all. In fact, occasional consumers are less likely to buy a functional extra virgin olive oil, while skeptical consumers attach less importance to functional properties and quality attributes. This last result can be reasonably explained by the strong influence of tradition on eating habits for a large number of Italian consumers, who prefer olive oil as it is, without any additive, functional components or flavor enhancers.

## 5. Concluding Remarks

This study evaluated consumers’ attitudes and willingness to pay an additional premium price for vitamin-enriched olive oil by using both a multivariate statistics and econometric approach. The descriptive outcome indicated that more than 60% of respondents showed an interest in paying extra for a vitaminized olive oil. However, a different additional price threshold was detected, which suggests that olive oil can be further differentiated in order to meet the demands of consumers interested in the health properties of foods. Regarding the first research question, it was addressed by initially obtaining the factorial dimensions to be used in an OLS regression in combination with socio-demographic data. The analysis showed the factors that drive consumers toward vitamin-enriched olive oil; specifically, age among the socio-demographics characteristics, belief related to disease prevention and extrinsic factors, and purchasing behavior among the factorial dimensions. The second research question was addressed by using the k-mean cluster multivariate approach where the principal components (PCs) in combination with socio-demographic characteristics were associated to the interest in functional vitaminized olive oil. We found that female gender and high levels of education were the variables associated with consumers’ interest in the functional properties of olive oil.

This study has several implications at the business level, and provides the olive oil industry, policy makers and academics with insights into consumer preferences towards healthier attributes of EVOO.

Concerning the importance among scholars, to the best of our knowledge, this is a first attempt to evaluate consumers’ interest in vitamin-enriched olive oil. Therefore, this study enhances the current debate about olive oil consumers, highlighting the key drivers and socio-demographic predictors for specific interest in functional properties of olive oil.

With regard to producers, the production of vitamin-enriched olive oil could satisfy the interest of consumers, leading to the identification of a new niche in the market. So, based on our results, olive oil can be further differentiated to satisfy the interest of healthy consumers.

Although the production of such olive oil is an economic and technological challenge for companies, they can evaluate the feasibility of producing for a wider range of consumers. The findings of this study may have consequences as they provide early evidence of consumers’ inclination for fortified olive oils. This data could be a valuable starting point for developing new marketing approaches, which can lead to the establishment of competitive advantages for the olive oil industry.

The study’s limitations are ascribable mainly to the nature of our sample, which is national and convenient; this implies that a certain prudence is required in extending the results to a European consumer. Finally, this study only provides a partial explanation of the drivers that move consumers toward the product evaluated, considering that other constructs could be adopted.

However, this study represents a first attempt to assess the feasibility of an enriched olive oil to be marketed by assessing consumer interest in such a product, and gives some preliminary insights to academics and producers. Further studies should be conducted to better understand and confirm what this article proposes. Thus, the role of consumer knowledge, the influence of lifestyle and psychological predictors in choosing vitamin-enriched olive oils could be further investigated.

It should also be possible to assess the interest in such a product in other countries or even to develop a cross-country study, and to use other methodological approaches such as choice or conjoint experiments.

## Figures and Tables

**Table 1:** Socio-demographic characteristics of the sample (*n* = 588).

Variables	Categories	Frequency	Percent
Gender	Male	229	38.95
	Female	359	61.05
Age cohort	Younger Generations (18–37)	398	67.69
	Generation X (>38)	190	32.31
Education	Elementary and middle school	72	12.24
	High School	241	40.99
	University	275	46.77

**Table 2:** Results from the exploratory factor analysis (EFA) *.

Intrinsic Factors	Factor Loading	Extrinsic Factors	Factor Loading
Safety	0.7280	Brand	0.7269
Quality	0.7598	Price	0.7058
Color	0.7406	Label indications	0.8181
Taste	0.7790	Olive provenance	0.6637
Aroma	0.7743	Quality certification	0.7459
Consistency	0.6378	Traceability	0.7175
**Beliefs**		**Perception**	
Olive oil has a lower fat content	0.7920	Healthiness	0.8450
Olive oil contains no additives	0.7917	Higher digestibility	0.8019
Olive oil helps keep cholesterol down	0.7833	Nutritional properties	0.8701
Olive oil does less harm than seed oil	0.7080	Culinary traditions	0.7853
**Organoleptic qualities**		**Purchasing behavior**	
Spicy	0.8022	Regularly purchase	0.8802
Bitter	0.8213	Often purchase	0.8756

* Rotated factor loading varimax blank (0.6).

**Table 3:** OLS regression results.

Variables	Coef.	Robust Standard Error	*p*-Value
Age	−0.243	0.056	<0.001 ***
Gender	−0.509	1.314	0.699
Education	−0.771	1.016	0.448
Disease Prevention	1.739	0.529	<0.001 ***
Perception	−1.139	0.646	0.078 *
Intrinsic factors	−0.084	0.601	0.889
Extrinsic factors	1.191	0.657	0.070 *
Beliefs	0.344	0.671	0.608
Organoleptic qualities	0.742	0.666	0.266
Purchasing behavior	1.232	0.703	0.080 *
Constant	16.935	4.788	0.000

OLS, ordinary least squares regression; Coef., Coefficient. Note ***, * indicate significance at 0.01, and 0.10 levels, respectively.

**Table 4:** Results of cluster analysis using k-means method.

Variables	Cluster 1 Aware Healthy Consumers (*n* = 225)	Cluster 2 Skeptical Consumers (*n* = 98)	Cluster 3 Occasional Consumers (*n* = 128)	Cluster 4 Informed Quality Seekers (*n* = 137)	*p*-Value
Perception	0.217	−0.556	0.144	−0.093	<0.001
Intrinsic factors	0.181	−1.609	0.080	0.778	<0.001
Extrinsic factors	0.096	−0.491	0.159	0.045	<0.001
Beliefs	−0.087	−0.388	0.193	0.239	<0.001
Organoleptic qualities	0.721	−0.624	0.294	−1.012	<0.001
Purchasing behavior	0.564	0.244	−1.012	0.337	<0.001

**Table 5:** Socio-demographic characteristics of clusters and interest in functional extra virgin olive oil.

Variables	Categories	Cluster 1	Cluster 2	Cluster 3	Cluster 4	Chi-Square
Gender	Male	0.31	0.48	0.45	0.39	11,351 ***
	Female	0.69	0.52	0.55	0.61	
Age cohort	Younger Generations	0.68	0.67	0.77	0.58	10,083 **
	X Generation	0.32	0.33	0.23	0.42	
Education	Primary and middle school	0.10	0.9	0.10	0.20	15,425 **
	High School	0.36	0.49	0.42	0.42	
	University	0.53	0.48	0.54	0.39	
Interest in functional olive oil	No	0.24	0.33	0.38	0.36	9140 **
	Yes	0.76	0.67	0.62	0.64	
Interest in vitamin-enriched olive oil	No	0.27	0.34	0.42	0.39	10,559 ***
	Yes	0.73	0.66	0.58	0.61	

Note ***, ** indicate significance at 0.01, and 0.05 levels, respectively.

## Data Availability

The data that support the findings of this study are available on request from the corresponding author (D.S.)
